# Oral Photoprotection: Effective Agents and Potential Candidates

**DOI:** 10.3389/fmed.2018.00188

**Published:** 2018-06-26

**Authors:** Concepción Parrado, Neena Philips, Yolanda Gilaberte, Angeles Juarranz, Salvador González

**Affiliations:** ^1^Department of Histology and Pathology, University of Málaga, Málaga, Spain; ^2^School of Natural Sciences, Fairleigh Dickinson University, Teaneck, NJ, United States; ^3^Dermatology Service, Hospital Miguel Servet, Zaragoza, Spain; ^4^Dermatology, Hospital Universitario Miguel Servet, Zaragoza, Spain; ^5^Biology Department, Instituto Ramón y Cajal de Investigación Sanitaria, Universidad Autónoma de Madrid, Madrid, Spain; ^6^Medicine and Medical Specialties Department, Instituto Ramón y Cajal de Investigación Sanitaria, Alcalá University Madrid, Madrid, Spain

**Keywords:** oral photoprotection, oxidative stress, dietary botanical, photodamage, photocarcinogenesis

## Abstract

Electromagnetic radiation in the ultraviolet, visible, and infrared ranges produces biologic effects in humans. Where some of these effects are beneficial, others are harmful to the skin, particularly those stemming from ultraviolet radiation (UVR). Pharmacological photoprotection can be topical or systemic. Systemic photoprotection is often administered orally, complementing topical protection. New and classic oral agents (e.g., essential micronutrients as vitamins, minerals, polyphenols, carotenoids) are endowed with photoprotective and anti-photocarcinogenic properties. These substances bear the potential to increase systemic protection against the effects of electromagnetic radiation in the UV, visible, and infrared ranges. Protective mechanisms vary and include anti-oxidant, anti-inflammatory, and immunomodulatory effects. As such, they provide protection against UVR and prevent photo-induced carcinogenesis and aging. In this review, we present state of the art approaches regarding the photoprotective effects of vitamins and vitamin derivatives, dietary botanical, and non-botanical agents. A growing body of data supports the beneficial effects of oral photoprotection on the health of the skin. More studies will likely confirm and expand the positive impact of oral dietary botanicals as complementary measures for photoprotection.

## Introduction: photoprotective agents

Sunscreen-based photoprotection is a major part of the first line of prevention to combat photoaging and skin cancer. Topical photoprotection is usually carried out by applying a thin ultraviolet radiation (UVR)-absorbing layer on the skin before sun exposure. Despite the incorporation of new technology and innovative approaches in topical photoprotection, inadequate use, and lack of optimization still limit usefulness of sunscreens. Topical sunscreens also have intrinsic limitations, among them, chiefly short half-life on the skin, which highlights the need for frequent reapplication, a lack of systemic efficacy, and potential side effects (1, 2). Despite the widespread use of sunscreens, sunburn remains commonplace.

Conversely, oral photoprotectors do not directly protect the skin against the damage induced by high energy photons; therefore, they are not very effective against the erythema and other deleterious effects caused by the sun. However, they do possess several advantages, mainly their ease of use. Also, their efficiency is not altered by external conditions, their half-lives can be determined pharmacologically, and their effects do not depend on the degree of absorption through the skin. The ideal photoprotective agent would be an oral photoprotector with cutaneous affinity. The overarching idea is that oral photoprotective agents need to provide uniform protection of the skin to be useful in the primary prevention of skin cancer and photoaging (1).

These oral photoprotective products usually contain one or more active principles that activate different mechanisms of photoprotection, especially those related to their anti-oxidant actions (1, 3). These substances act by increasing the anti-oxidant efficacy of the body following the loss of endogenous anti-oxidants after UVR exposure. UV radiation induces DNA damage, triggers inflammatory phenomena, and promotes tumor growth. It also contributes to aging through alterations in collagen remodeling and mitochondrial deletion. Most of these detrimental effects are mainly mediated by oxidative stress (2). Some of these substances also reduce UVR-induced immunosuppression.

The following sections provide an update of state of the art regarding the properties of oral photo protectors.

## Vitamin derivatives with anti-oxidant properties

### Single vitamins (Table 1)

#### Carotenoids

Carotenoids are pigments existing in a wide variety of vegetables and fruits, especially in tomatoes. They also appear in considerable amounts in human plasma and tissues. However, carotenoids are exclusively synthesized by plants. Hence those appearing in animals and humans have been acquired through the diet. Carotenoids decrease reactive oxygen species (ROS) in aerobic metabolism (29).

**Table 1:** Photoprotective effects of vitamins and their molecular targets.

**UV effects tissue/cellular/molecular target**	**Compound(s)**	**Results**	**Models**	**References**
Erythema	β-carotene	Slight increase in MED/ *Min. protection/≥10 weeks doses high ≥12 mg carotenoids/ day*	Human	(4, 5)
	Lycopene	Decreases erythema	Human	(6, 7)
Oxidative stress	Lycopene	Inhibits HO-1	Human	(8)
	Astaxanthin	Inhibits reductions SOD, GSH	*in vitro*	(9)
DNA damage	Lycopene	Inhibits mtDNA deletion	Human	(7)
Inflammation	Lycopene	Inhibits ICAM-1 expression	Human	(8)
	Astaxanthin	Inhibits MIF, IL-1β, TNF-α expression	*in vitro*	(10)
		Decreases masts cells	Mice	(11)
		Sustains trans-UCA levels	Mice	(10)
	Lutein/Zeaxanthin	Suppresses skin edema	Mice	(12)
		Decreases masts cells number	Mice	(13)
	Lycopene β carotene, *Lactobacillus Johnsonii*	Inhibits PMLE	Human	(14)
Immuno-suppression		–	–	–
Photo carcinogenesis	Lycopene	Inhibits skin tumor formation	Mice	(15)
	Astaxanthin	Inhibits apoptosis	*in vitro*	(10)
	Lutein/Zeaxanthin	Decreases BrdU + epidermal cells	Mice	(12)
		Decreases PCNA + epidermal cells	Mice	(12)
		Increases tumor-free survival time	Mice	(13)
		Inhibits tumor volume and multiplicity	Mice	(13)
UV-ECM damage	Lycopene	Inhibits MMP-1	Human	(7, 8)
	Lutein/Zeaxanthin	Inhibits MMP-1, MMP-7. Stimulate TIMP-2	*in vitro*	(16)
		Inhibits MMP-13	Mice	(10)
		Decreases overexpression of HO-1, ICAM-1, MMP1 genes	Human	(8)
	Lycopene, β-carotene, *Lactobacillus johnsonii*	Inhibits MMP-1	Human	(14)
**UV effects tissue/cellular/molecular target**	**Interventions**	**Results**	**Models**	**References**
Erythema	Vit. E+ Carotenoids	Suppression/decrease erythema	Human	(17, 18)
	Vit. C+Vit. E	Increases MED	Human	(19)
Oxidative stress	Vit E+ Carotenoids	Decreases levels of lipoperoxide	Human	(18)
DNA damage	Nicotinamide	Prevents depletion of cellular NAD+	*in vitro*	(20)
		Inhibits PARP-1	*in vitro*	(20)
		Inhibits CPD and 8oxoG	Human	(21)
			*in vitro*	(22)
	Vit. D	Reduces thymine dimers	Human	(23)
			Mice	(24)
		Inhibits CPD	*in vitro*	(25)
Inflammation	Vit. D	Inhibits NO products	*in vitro*	(23)
		Decreases edema and epidermal vesiculation	Human	(26)
		Decreases TNF-α and iNOS	Human	(26)
		Induces overexpression of ARG1	Human	(26)
		and genes involved in skin repair		
Immuno-suppression	Nicotinamide	Prevents suppression of Mantoux reactions	Human	(27)
	Vit. D	Reduces CHS response	Mice	(24)
Photo carcinogenesis	Nicotinamide	Reduces AK by 29%	Human	(21)
		Lowers the rate of new NMSC and AK	Human	(28)
	Vit. D	Increases keratinocyte survival	*in vitro*	(23)
		Increases p53 expression	*in vitro*	(23)
UV-ECM damage		–	–	–

*CHS, contact hypersensitivity; HO-1, Heme oxygenase-1; MED, minimal erythema dose; PMLE, polymorphic light eruption*.

About 50 variants of carotenes are present in a typical human diet and, of these, six are found mainly in the blood: α-carotene, β-carotene, zeaxanthin, lutein, β-cryptoxanthin, and lycopene. Of these, lycopene is the most efficient regarding anti-oxidant activity (30). *In vitro* and *in vivo* studies have revealed that carotenoids can suppress UVA and UVB-mediated ROS formation, thereby, preventing photoinactivation of anti-oxidant enzymes, lipid peroxidation, and induction of DNA damage caused by oxidative stress (30, 31).

##### Lycopene

Lycopene is the predominant carotenoid present in tomatoes and other vegetables and red fruits, except in cherries and strawberries. Lycopene, a polyunsaturated hydrocarbon (C40H56), is endowed with a very high anti-oxidant capacity quenching singlet oxygen (32). *in vitro* studies with human skin fibroblasts disclosed a reduction of UVB-induced lipid peroxidation by lycopene (33).

Several investigators have reported on the effects of lycopene in humans. Subjects treated with oral lycopene for 10 weeks had 40% less dorsal erythema formation in response to UVR compared to untreated subjects (6), as measured by chromametry (6). Similarly, an intervention study in which healthy women received tomato paste rich in lycopene during 12 weeks supplemented with olive oil suggested that lycopene exerted beneficial properties (7). Lycopene reduced matrix metalloproteinases 1 (MMP-1) overexpression and mtDNA 3,895-bp deletion produced by UVR. The mechanism proposed for lycopene relates to its anti-oxidant capacity, decreasing ROS production, and protecting cellular structures from UVR-induced damage (7).

A recent study described how 12-weeks of oral treatment with lycopene-rich tomato nutrient complex (TNC) inhibited the expression of UVB/A triggered genes that mediated skin's response to UV radiation (8). Lycopene inhibited UVA/B induced overexpression of heme oxygenase-1 (HO-1), an indicator of oxidative stress, and also decreased UVA/B induced overexpression MMP-1, a metalloproteinase involved in the breakdown of collagen and skin photoaging. Finally, lycopene curbed the expression of the inflammatory mediator ICAM-1, suggesting that this agent can inhibit the recruitment of leukocytes to the skin upon UVR-mediated damage and inflammation (8). Another recent study has shown that treatment of Skh-1 mice for 34 weeks with tomato-rich diet significantly decreased tumor induction by UVB irradiation compared to animals receiving a regular food (15). Moreover, the combination of lycopene with other carotenoids and *Lactobacillus johnsonii* also protected against UVA-induced polymorphous light eruption in human subjects (14).

The three clinical trials referenced above (6–8) had in common the duration of the treatment (12 weeks). However, they used different concentrations of lycopene and/or supplements, e.g., olive oil. Hence, it is not possible to properly correlate the doses with the observed effects. The anti-oxidant power of lycopene is well-proven regarding photoprotection, but there is not a consensus regarding the preventive dose required and the effect of combining it with other substances, highlighting the need for additional clinical research in the use of lycopene for oral photoprotection.

Beta (β)-carotene is a compound often administered for systemic photoprotection. However, studies demonstrating a protective effect of oral treatment with β-carotene against skin photodamage are scarce or revealed contradictory results. Intervention studies showed that a high intake of β-carotene decreased UVR induced erythema, but the efficacy of β-carotene depended on the dose and duration of treatment (31). Healthy volunteers receiving a supplement of β-carotene exhibited a slight increase of the threshold of minimal erythema dose (MED) (4). Similarly, partial protection against UVA and UVB radiation were observed in a study in which β-carotene was administered orally (5). Specifically, β-carotene reduced serum lipid peroxidation in a dose-dependent manner (5).

Regarding the effect of β-carotene in UVR-induced erythema, a placebo-controlled study showed that pretreatment with β-carotene diminished the intensity of erythema caused by sunlight (34). Similarly, oral administration of β-carotene in volunteers with Fitzpatrick's skin phototype II decreased the severity of UVR-induced erythema (35). Thus, in the supplemented group with β-carotene, Δα-values significantly decreased by 37.3% after 12 weeks of treatment compared to untreated group (35). In all the studies that documented some protection against UVR-induced erythema, the period for the supplementation was relatively long (~≥10 weeks) with high doses (~≥12 mg/day) (4, 5). This fact has raised concerns regarding the safety of administering such high doses of β-carotene. An epidemiological study suggested that high levels of β-carotene may have a deleterious effect in individuals at high risk of lung cancer, e.g., in smokers (more than a pack a day for 35 years) and asbestos workers. In these high-risk subjects β-carotene intake resulted in an enhanced risk of lung cancer compared to subjects bearing a lower risk of lung cancer (36). In a recent *in vitro* study published in 2018, the group of Lohan et al. has measured the anti-oxidant activity of β-carotene in a keratinocyte cell line (HaCaT) using electronic paramagnetic resonance spectroscopy and found that the anti-oxidant protection against UVR was achieved only with low doses of β-carotene whereas high doses were prooxidant (37).

However, based on long-term experience from the results obtained in the 1970s (4) and controlled trials (38) oral administration of β-carotene has been the treatment of choice to improve the photosensitivity of patients with erythropoietic protoporphyria. Photosensitivity was reported being reduced in ~80% of patients to allow them normal life activities (38). The doses recommended range from 30 to 90 mg/day for children and 60–180 mg/day for adults, to reach a maximum plasma level of 600–800 μg/dl. More recently, subcutaneous administration of afamelanotide, an analog of the α-melanocyte-stimulating hormone, that darkens the skin, has been proposed as a novel treatment for erythropoietic protoporphyria (39).

##### Xanthophylls

Xanthophylls include some other carotenoids, e.g., lutein, astaxanthin, and zeaxanthin, which all have been shown to prevent photodamage induce by sunlight (9).

##### Astaxanthin

Astaxanthin is a non-provitamin A carotenoid mainly found in fish and shellfish (10). It is endowed with an anti-oxidant effect more potent than other carotenoids, including β-carotene and exerts anti-oxidant benefits without having prooxidant side effects. Astaxanthin inhibits the production of lipid peroxides induced by UVA. *in vitro* experiments have indicated its anti-oxidant and anti-inflammatory activity. In human skin fibroblasts astaxanthin prevented UVA-induced alterations of superoxide dismutase (SOD) activity and the anti-oxidant glutathione (GSH) (40).

Furthermore, treatment with astaxanthin reduced UVB- or UVC-induced expression of macrophage migration inhibitory factor (MIF), interleukin-1 (β IL-1β), and tumor necrosis factor α (TNF-α) (41). Astaxanthin significantly inhibited UV-irradiation-induced apoptosis in HaCaT keratinocytes (10). Treatment with astaxanthin before and after irradiation with UVB and UVA (41) decreased MMP-1 expression (11). Also, astaxanthin inhibited the UVB-induced expression of activator protein AP-1 and reduced UVB-induced phosphorylation of several MAPK family members via AP-1 transactivation in human fibroblasts (11).

A recent study reported the beneficial effects of oral astaxanthin on skin photoaging prevention *in vivo* (10). In a mouse model, astaxanthin inhibited the UVA-induced decrease of pyroglutamic acid (PCA) and urocanic acid (UCA), which are the primary natural moisturizing factors in the epidermis (10). In this murine model, astaxanthin also inhibited UVA-induced expression of matrix metalloprotease 13 (MMP-13), which may underline its photoprotective effect against skin photodamage (10).

Beneficial effects of astaxanthin have been reported with regard to human skin aging by Chung et al. (42). The same group of investigators is conducting a clinical trial to determine the effects of supplementation with astaxanthin or isoflavone on skin elasticity, epidermal hydration, and changes the skin barrier integrity. However, the results of this study are yet to become available.

##### Lutein and zeaxanthin

The xanthophylls, zeaxanthin, and lutein stand for 20–30% of the total carotenoids present in human serum and 80–90% of the carotenoids in the human retina.

Zeaxanthin is equitably distributed among plants, accompanying other carotenoids. It is typical of corn (maize), and also many bacteria produce it. Lutein is found in many vegetables, such as green beans, spinach, or broccoli, although its color is masked by chlorophyll. Zeaxanthin and lutein and are found in the macula where they contribute to preventing macular degeneration (43).

Lutein also accumulates in the skin. Its anti-aging and anti-carcinogenic properties are based on its anti-oxidants and anti-inflammatory effects against UVR damage. In mice, dietary lutein supplementation decreased ROS generation following UVR exposure (44). Specifically, our group reported the beneficial effects of orally administered lutein and zeaxanthin against the deleterious effects of UVB radiation. In hairless SKh-1 mice, supplementation with 0.4% lutein plus 0.04% zeaxanthin decreased the UVB-induced acute inflammatory responses (12). These photoprotective effects also included lower numbers of bromodeoxyuridine and proliferating cell nuclear antigen (PCNA)-positive cells in the epidermis, reduced skinfold thickness, and lower number of mast cells in the skin following UVB irradiation (13). Regarding UVR induced photocarcinogenesis, we found that oral supplementation with lutein/zeaxanthin significantly increased tumor-free survival time, decreased the total tumor volume, and reduced tumor multiplicity in comparison with control animals (13). We also reported lutein's photoprotective effects in UV irradiated dermal fibroblasts and melanoma cells. Lutein improved membrane integrity, increased cell viability, and decreased elastin expression. Lutein also inhibited UVR-induced overexpression of MMP-1 and MMP-2 while stimulating the endogenous tissue metalloproteinase inhibitor TIMP-2 (16).

Recently a placebo-controlled, double-blinded, randomized, crossover study reported that orally supplemented lutein caused a significant reduction of the overexpression of HO and MMP-1 genes induced by UVA radiation (8). Since these genes are reliable indicators of oxidative stress and photoaging, these results suggest that lutein may protect against photodamage produced by solar radiation (8, 45).

#### Nicotinamide

Nicotinamide is an amide form of vitamin B3 and a precursor of the essential coenzymes such as nicotinamide adenine dinucleotide (NAD^+^) (21). Its primary dietary sources are liver, meats, yeast, legumes, nuts, green leafy vegetables, cereals, tea, and coffee (2, 21). It has been used to treat a variety of dermatological diseases such as atopic dermatitis and acne (2). Recent studies highlighted the role of nicotinamide, administered both orally and topically, as a chemopreventive agent against skin cancer. Its anti-cancer function is due to its corrective action toward UVR-induced DNA damage, also preventing immunosuppression.

Nicotinamide promotes genomic stability and DNA repair. NAD^+^ is a substrate for poly-ADP-ribose polymerase 1 (PARP-1), which detects DNA damage (21). Nicotinamide prevents the depletion of cellular NAD^+^ levels in response to exposure to UVR (20). Therefore, nicotinamide supplementation may prevent the progression of actinic keratosis (AK) to malignant squamous cell carcinoma (27, 46–48). In a very recent study, we found that niacin and its derivatives significantly promoted the expression of elastin, fibrillin-1, and fibrillin-2 in non-irradiated, and UVA-radiated fibroblasts, and directly inhibited MMP or elastase activity (49).

Nicotinamide also prevents UVR-induced intracellular depletion of adenosine triphosphate boosting cellular energy and enhancing DNA repair in HaCaT cells (20). In human, exposure to UV solar-simulated radiation triggered the formation of cyclobutane pyrimidine dimers (CPDs) and 8-oxo-7,8-dihydroguanine (8oxoG). Nicotinamide reduced CPDs and 8oxoG formation both *in vivo* and *in vitro* (22, 48).

Nicotinamide also inhibits the activity of sirtuins, which are NAD^+^ dependent enzymes. Sirtuins play a mandatory role in cellular responses to environmental stress (47). Its effect on various transcription factors, including p53, contributes to the regulation of cell survival. Sirtuin expression is triggered by UV irradiation and is upregulated in AK and squamous cell carcinoma, suggesting that sirtuins may be associated with early stages of skin cancer. In healthy volunteers, using the Mantoux model of skin immunity, oral nicotinamide significantly reduced UVR-induced immunosuppression (27).

A potential protective role of nicotinamide in photocarcinogenesis has been reported in non-melanoma skin cancer (NMSC). In two clinical trials, nicotinamide decreased the incidence of NMSC and AK (28, 50). Immune-competent volunteers with ≥4 palpable AKs (face, scalp, and upper limbs) were treated with 500 mg of nicotinamide once a day for 4 months. Nicotinamide resulted in a relative reduction of 29% in AK count in the active treatment group compared with the placebo group (50). Along the same line, a double-blind, phase III controlled trial revealed that patients have suffered two or more NMSC and treated with nicotinamide had 23% lower rates of new NMSC and 11% less actinic keratoses than placebo-treated patients (28). This chemopreventive effect only persisted with continuous treatment (28). Adverse effects of nicotinamide were rare, and unlike niacin, nicotinamide is not a vasodilator. The administered dose of nicotinamide was 500 mg twice daily, and no more significant benefits were observed with higher doses (28).

However, a controversy emerged in response to a publication by Yelamos et al. (51). The authors concluded that nicotinamide may reduce the number of AKs, but only the less aggressive types, whereas in overall it may increase the rate of more aggressive types. The effect of oral nicotinamide as a chemopreventive agent against skin cancer may be due to its ability to enhance DNA repair and prevention of photoimmunosuppression (52). Additional clinical trials with larger cohorts of patients and more extended follow-up periods are necessary to solve this apparent controversy.

#### Vitamin D

Vitamin D3 (cholecalciferol) is obtained mainly from two essential sources: diet (10%) and endogenous production by photochemical conversion from 7-dehydrocholesterol in the epidermis (90%). Endogenous synthesis is induced by exposure of the skin to ultraviolet B (UVB) radiation. The skin is also a target tissue for the active form of Vitamin D3 [calcitriol, 1,25 (OH) 2D3] and other biologically active metabolites of vitamin D3 (53). 25-hydroxyvitamin D_3_ and 1,25-dihydroxy vitamin D_3_ are also produced by keratinocytes and macrophages (54, 55). Vitamin D3 modulates inflammatory, immune responses and carcinogenesis (56, 57). Vitamin D3 decreases the inflammatory response by negatively regulating pro-inflammatory mediators, including TNF-α and nuclear factor-κB (NF-κb) one of the essential factors in inflammation. Vitamin D3 also decreases cyclooxygenase 2 (COX2), with the consequent decrease in prostaglandin levels (56, 58). The action of this vitamin has been reported in a mouse model of chemically-induced skin injury where a single dose of it attenuated the inflammatory response by inhibition of iNOS protein (or *NOS2)* gene and TNF-α protein (or *TNFA* gene) (59).

Similar to many other steroid hormones, 1,25 (OH) 2D3 exerts its action primarily through two signal transduction pathways: the classical genomic and the non-genomic pathway. The non-genomic effects depend on the levels of intracellular calcium whereas the genomic effects are mediated by the vitamin D receptor (VDR) (56). Recent findings support the role of VDR as a tumor suppressor in the skin. The anti-tumor effects of VDR are mediated, at least in part, by its interaction with p53 gene in response to UVR-induced DNA damage. Several studies have proposed that vitamin D3 also regulated the Hedgehog (Hh) signaling pathway. The Hh signaling pathway has been related to basal cell carcinomas (60). In the skin, keratinocytes, melanocytes, fibroblasts, and Langerhans cells express the VDR (53).

*In vitro* and *in vivo* studies showed that treatment with 1,25(OH)2D3 increased the survival of keratinocytes post-UVR compared to vehicle (23, 25). 1,25(OH)2D3 caused a significant reduction in the formation of CPD (23, 25) and increased the expression of p53 in keratinocytes (23). Moreover, dietary supplementation of 25(OH)D3 reduced UVB mediated contact hypersensitivity (CHS) response in C57BL/6 mice, a murine model with high susceptibility to UVB-induced systemic immunosuppression compared to mice with a deficient diet of this compound. Similarly, there was also a reduction in CPDs and inflammation in the animals supplemented with 25(OH)D3 (24).

In a recent clinical trial, participants were treated with a single oral dose of vitamin D3 (cholecalciferol) 1 h after UVR exposure. After irradiation, the human skin showed histological damage, including edema formation and epidermal vesiculation, which was diminished in a vitamin D3 dose-dependent manner. Skin expression of TNF-α and inducible isoform of nitric oxide synthase (iNOS) was lower in participants receiving Vitamin D3 as compared in those receiving placebo (26). In the same study, the genetic profile of the participants was evaluated independently of the treatment. Two distinct groups were identified. Group 1 was characterized by a lower expression of arginase (ARG)-1, which favors tissue repair and inhibits inflammation. Group 2 was marked by overexpression of ARG1 and genes involved in the restoration of the skin barrier. When assessing the treatments given in both groups, it was found that in group 2 all the participants had received a high dose of vitamin D3 and no participant received placebo. As a result, most participants in group 1 received placebo, and some received different doses of vitamin D3. Group 2 was identified as vitamin D3 responders of and group 1 vitamin D3 non-responders. The Vitamin D3 non-responders (group 1) had overexpression of proinflammatory genes, for example, IL-1α, MMP-1, and MMP3. In contrast, vitamin D3 responders (group 2) did not exhibit this characteristic. Similarly, IL-6 was activated significantly in patients who did not respond to vitamin D3. The authors of this trial proposed that a single oral dose of vitamin D3 rapidly mitigated the local UVR-induced inflammatory response in sensitive individuals. They also found that vitamin D3 responder showed a marked decrease in facial redness after an experimental sunburn, less evidence of epidermal damage and a lower expression of proinflammatory markers in the skin. As outlined above, the vitamin D3 responders had a genetic profile of overexpression of cutaneous barrier repair genes. Since the dose of vitamin D used had no adverse effects, and the calcium levels remained normal, the investigators of the study concluded that a single dose of high vitamin D3 could be of clinical use to prevent photodamage (26). Growing evidence sustains the perception that vitamin D pathway is relevant for photocarcinogenesis and that the pharmacological action of vitamin D, 1,25 (OH)2D3 and its analogs represent an advantageous new strategy for the prevention of UVR-induced damage (26, 61).

#### Vitamin C

Vitamin C given alone does not prevent the deleterious effects of UVR in the skin (19). Consequently, dietary supplementation of vitamin C (500 mg/day) for 8 weeks did not affect the UVR-induced erythema response. Furthermore, vitamin C supplementation in this group of healthy volunteers produced a paradoxical effect since the content of malonaldehyde and thiol-containing, and glutathione-binding proteins were reduced in the skin (62).

#### Vitamin E

Skin exposure to UVR depleted the cutaneous levels of vitamin E (alpha-tocopherol), implying that vitamin E is efficiently quenching ROS in UVR skin exposure (63). However, there is no evidence about the beneficial effects of oral vitamin E supplementation in the reduction of UVR-induced skin damage (19, 64). Conversely, supplementation of other components with vitamin E does show some benefit (see below) (19, 65). Likewise, MED was not changed by 400 IU of oral vitamin E alone after administration of 1 and 6 months (64). A side-by-side comparison of the effects of β-carotene (15 mg/day) vs. vitamin E (400 IU/day) for 8 weeks revealed that only vitamin E decreased the skin malondialdehyde concentration. However, neither β-carotene nor vitamin E changed other measures of oxidation UVR-exposed skin (65).

### Vitamin mixtures (Table 1)

Several groups or researchers from pharmaceutical companies and academic institutions developed mixtures of anti-oxidant. Such combinations were found to possess slight photoprotective, but they need to be administered at high doses and for an extended period of time to obtain a modest degree of protection (17).

#### Carotenoids and vitamin E

Supplementation with carotenoids and vitamin E for 3 months provided minimal photoprotection. Erythema was diminished with carotenoids (decreased of Δα erythema values by 34.5% after 8 weeks of treatment), but erythema suppression was amplified by the combination of carotenoids and vitamin E (decreased Δα-values by 43.19%) (66). An anti-oxidant complex with vitamin E with b-carotene and lycopene (with additional selenium and RRR-a-tocopherol) also protected against UVR-induced skin damage (18). This anti-oxidant compound increased the actinic erythema threshold, increasing MED by 20%. The anti-oxidant complex also decreases the p53 expression and lipoperoxide levels (18).

On the other hand, mixtures of anti-oxidants containing carotenoids (b-carotene and lycopene), vitamins C and E, selenium, and proanthocyanidins revealed no significant change in light sensitivity. However, they showed a decrease in UVR-dependent expression of MMP-1 (67).

#### Vitamin C and vitamin E

Based on the rationale that supplementation of vitamin C regenerates cutaneous vitamin E from its radical form, the combination of both was thought to act synergistically. In this regard, different studies investigating supplementation with a mixture of vitamin C and vitamin E has been reported. In a retrospective human study, the combination of ascorbic and a-tocopherol during 7 weeks increased the MED by 77.6% (from 103 ± 29 mJ/cm^2^ before supplementation to 183 ± 35 mJ/cm^2^) (19). Similarly, during 1-week of oral intake of C and vitamin E increased protection of skin against UVR, as it increased MED by 21% (68). In another study, the same group of investigators studied the administration of ascorbic acid and α-tocopherol over a period of 12 weeks, which increased MED by 41% and decreased UVR-induced CPD (69). In another study of the same group of investigators ascorbic acid and α-tocopherol were given over a period of 12 weeks, and they found an increase of the MED by 41% and decrease of UVR-induced CPD (72). The addition of 3-methoxy-4-hydroxycinnamic acid (ferulic acid) did improve the stability of the combination of both vitamins. 1% alpha-tocopherol and vitamins (C+E) provided doubled photoprotection to solar-simulated radiation of skin as measured by both sunburn cell formation and erythema. Inhibition of apoptosis with the combination of both vitamins and ferulic acid was associated with inhibition of UVR-induction of caspase-3 and caspase-7 (70). The mechanism of this synergy does not seem to be clear, but it could be due to the power of ascorbate to produce a reduction of tocopherol, by transferring free radicals captured to the medium. On the skin, these free radicals are neutralized by other anti-oxidant systems.

## Dietary non-botanicals (Table 2)

### ω-3 polyunsaturated fatty acids

Omega-3 polyunsaturated fatty acids have been considered to treat skin conditions related to UVR exposure. They modestly decreased the appearance of sunburn cells and inflammation upon UVR treatment as well as long-term effects of UVA exposure (75). Omega-3 fatty acids were effective in the treatment of Hydroa vacciniforme (HV), a rare photodermatosis (76). Their main limitation as an oral photoprotector is that a relatively high dose is needed for the effect, often being higher than the gastric tolerance threshold. Another drawback is their unpleasant taste.

**Table 2:** Photoprotective effects of non-botanical compounds and their molecular targets.

**UV effects tissue/cellular/molecular target**	**Compound(s)**	**Results**	**Models**	**References**
Erythema		–	–	–
DNA damage		–	–	–
Inflammation	**Probiotics** *Lactobacillus johnsonii* (La1)	Increases IL-10	Mice	(71)
Immuno-suppression	**Probiotics**			
	La1	Suppresses CHS reaction	Mice	(71)
	*Lactobacillus rhamnosus* GG	Increases number of activated dendritic cells in the mesenteric lymph nodes	Mice	(72)
	La1	Facilitates recovery of eLc	Human	(73)
	La1+carotene	β-decreases PMLE	Human	(74)
	La1+carotene	β-decreases CD45+ dermal inflammatory cells.	Human	(74)
Photo carcinogenesis	**Probiotics**			
	*Lactobacillus rhamnosus* GG	Delays appearance of skin tumors	Mice	(72)
UV-ECM damage		–	–	–

*eLc, epidermal Langerhans cells. EPP, erythropoietic protoporphyria; PMLE, polymorphic light eruption; CHS, contact hypersensitivity*.

### Probiotics

Probiotics are living microorganisms that regulate the immune system of the gut and defend it against inflammatory and infectious diseases.

In hairless Skh-1 mice exposed to UVR, supplementation with *L. johnsonii* NCC 533 (La1) conferred protection against the UVR-induced suppression of CHS and increased IL-10 serum levels (71). Oral administration of *Lactobacillus rhamnosus GG* delayed the onset of skin tumors in mice chronically irradiated with UV radiation. A significant improvement of the immune response was found in the small intestine of *Lactobacillus rhamnosus GG* treated mice with an increase of activated dendritic cells (72).

In humans, La1 supplementation accelerated the recovery of the function of Langerhans cell after UVR exposure in humans (73). Also a human dietary supplement combining La1 with nutritional doses of β-carotene prevented sunburn and sun intolerance in most of the study participants, protecting against the development of UVA-induced polymorphous light eruption (74).

The role of probiotics in photoprotection is promising, but it is necessary to carry out more extensive clinical trials before making a definitive recommendation on the use of probiotics as oral photoprotective agents (77).

### Idebenone

Idebenone, a lipophilic coenzyme Q10 analog, has a relatively high penetration into the skin upon topical administration. Its efficacy as an oral photoprotector has not been studied, but its oral administration increased the expression of nerve growth factor (NGF), and it is beneficial in patients with Leber's hereditary optic neuropathy (78).

## Dietary botanicals (Table 3)

This general term includes anti-oxidant and anti-inflammatory polyphenols found in vegetable foods. In the last decade, plenty of interest has emerged regarding the possible health benefits of polyphenols as anti-oxidants. The main classes of polyphenols are phenolic acids, flavonoids, stilbenes, and ligands. Flavonoids represent the most significant natural anti-oxidants present in dietary botanicals. Due to their chemical nature, which contains phenolic rings, they can absorb free radicals to form phenoxy radicals (1, 3). There are different subfamilies of flavonoids owing their chemical structure, which include flavanonols, aurones, isoflavones, flavonols, flavones, and anthocyanins. On the following pages, we summarize the main findings regarding several subclasses of polyphenols as oral photoprotective agents.

**Table 3:** Photoprotective effects of Botanical compounds and their molecular targets.

**UV Effects Tissue/cellular/molecular target**	**Compound(s)**	**Results**	**Models**	**References**
Erythema	Green tea	Decrease erythema	Human	(79)
	Polyphenols	*Green tea catechins + Vitamin C*		
	Cocoa extract	Decreases erythema/Increases MED	Human	(80, 81)
	PL	Decreases erythema/Increases MED	Human	(82, 83)
				(84)
	Citrus + Rosemary	Increases MED	Human	(85, 86)
Oxidative stress	PL	Inhibits lipid peroxidation	Human *in vitro*	(87, 88)
		Enhances anti-oxidant plasma capacity	Mice	(89)
	Pomegranate	Inhibits lipid peroxidation Inhibits hydrogen peroxide	Mice	(90)
DNA damage	Green tea polyphenols	Decrease CPD	Mice	(91, 92)
		Increase NER genes	Mice	(92)
	PL	Reduces 8oxoG	Mice	(93)
		Reduces number of DNA mutations	Mice	(93)
		Inhibits CPD	Mice	(93)
			Human	(83, 94)
		Reduces common mitochondrial deletions	Human	(95)
	Pomegranate	Reduces 8oxoG	Mice	(90)
		Inhibits CPD	Mice	(90)
	Forskolin	Improves NER	*in vitro*	(96)
Inflammation	Green tea polyphenols	Induce the secretion of IL-12	*in vitro*	(97)
		Inhibit AP-1 NF-κB	Mice	(98)
		Inhibit 12-LOXE metabolites	Human	(79)
	PL	Inhibits TNF-α, iNOS, AP-1 NF-κB expression	*in vitro*	(99)
		Increases IL-10 expression	*in vitro*	(100)
		Inhibits leukocyte extravasation	Mice	(101)
		Decreases neutrophil and macrophages	Mice	(93)
		Decreases mast cells	Human	(83, 102)
		Inhibits COX-2, PGE2	Mice	(93)
			Human	(94)
	Pomegranate	Inhibits COX2, NF-κB;	Mice	(90)
Immuno-suppression	PL	Inhibits *trans*-UCA isomerization	*in vitro*	(103)
		Inhibits glutathione oxidation	Mice	(101, 104)
		Prevents eLC depletion	Mice	(101)
			Human	(82, 83, 105)
		Reduces PMLE reaction Improves subjective symptoms of PMLE	Human	(106, 107)
				(108)
Photo carcinogenesis	Green tea Polyphenols	Decrease keratinocyte apoptosis	*in vitro*	(97)
		Protect against the development of NMSC (tumor incidence, tumor multiplicity, tumor size)	Mice	(109)
		Reduce CD31 and VEGF expression	Mice	(109)
		Reduce tumor development (number of tumors, tumor volume)	Mice	(91)
		Inhibit PCNA + epidermal cells	Mice	(109)
	PL	Increases the number of p53(+) cells	Mice	(89, 93)
		Delays skin tumor development	Mice	(89)
		Increases the clearance of AKs Decreases the recurrence rate of AKs	Human	(110)
		Increases MED in familial MM	Human	(84)
		Inhibits epidermalcell proliferation	Human	(83, 94)
		Decreases PCNA, Cyclin D1 expression	Human	(94)
	Isoflavones (Genistein)	Inhibit skin tumor formation	Mice	(111)
	Pomegranate	Inhibits PCNA expression	Mice	(90)
	Resveratrol	Inhibits NF-kB expression	*in vitro*	(96)
		Inhibits TGF-beta expression	Mice	(112)
		Decreases tumorigenesis	Mice	(112)
	Forskolin	Reduces sunburn cells	*in vitro*	(113)
UV-ECM DAMAGE	Green tea polyphenols	Reduce MMP-2 MMP-9 Enhance TIMP	Mice	(109)
	Cocoa extract	Attenuates skin wrinkling Decreases cathepsin G Improves Serpin B6c	Mice	(114)
	PL	Increases types I, III, and V collagen	*in vitro*	(115)
		Inhibits MMP-1	*in vitro*	(88, 115)
		Increases TIMP	*in vitro*	(115)
			Mice	(93)
		Decreases MMP1 after VIS-IR radiation	Human	(116)

*NER, Nucleotide excision repair*.

### Green tea polyphenols (GTPs)

The primary anti-oxidant moiety of green tea (*Camellia sinensis*) is a mixture of polyphenols (frequently referred to as catechins or green tea polyphenols, GTPs). The major catechins of green tea are epigallocatechin-3-gallate (EGCG), epicatechin-3-gallate (ECG) and epicatechin (EC), epigallocatechin (EGC). EGCG constitutes ~40% of total GTPs at the source (green tea leaves) (117). Numerous studies have demonstrated that tea catechins are efficient scavengers of ROS. Besides their anti-oxidant activity, catechins exhibit a modulating effect on inflammatory and immunomodulation responses playing an essential role in host defense against tumor development and progression (117). Interestingly, green tea confers protection against skin cancer in mice induced by UVA and UVB radiation (118).

Following standard photocarcinogenesis protocols using hairless mice, oral administration of GTPs in drinking water resulted in significant protection against the development of NMSC regarding tumor multiplicity, tumor size, and tumor incidence (percentage of mice with tumors) compared to no-GTPs-treated UVB-irradiated mice (109). Also, hairless mice receiving oral GTPs reduced UVB-induced overexpression of MMP-2 MMP-9 and enhanced expression of tissue inhibitor of MMPs. Oral GTPs administration also reduced UVB-induced expressions of vascular endothelial growth factor (VEGF), CD31 and inhibited expression of PCNA, resulting in decreased apoptosis and lower activation of the mitogen-activated protein kinase (MAPK) pathway (119).

GTPs also act against photoaging by preventing of UVR-induced activation of inflammatory transcription factors AP-1 and NF-κB (98, 120). When added in cultured human keratinocytes before, and/or after UVB irradiation, EGCG inhibited AP-1 activity (121).

It is precisely established that IL-12 deficiency increases the UVR-induced inflammatory response and decreases DNA repair in response to UVR-induced damage (91). In keratinocytes and human living skin equivalent models, GTPs induced the secretion of IL-12 and decreased keratinocyte apoptosis caused by UVB radiation (97). GTPs in drinking water significantly reduced the UVB-induced tumor development (volume and number of the tumors) and the number of CPD^+^ cells in wild-type mice but did not affect IL-12-deficient mice (91). These data suggest that GTPs prevent the photocarcinogenesis primarily by a mechanism that involves IL-12.

GTPs also increase the expression of nucleotide excision repair (NER) genes. Oral GTPs in mice had in the skin a reduced the number of CPD^+^ cells, showing thus faster repair of UVR-induced DNA damage. Also, GTPs decreased the migration of CPD^+^ cells to draining lymph nodes (92). Moreover, green tea catechins (GTC) reduced UVR-induced inflammation and protected from UVR-radiation immunosuppression and photo-carcinogenic effects in rodent models, but human studies are scarce and controversial.

Rhodes and colleagues examined the ability of GTPs to protect the skin from the effects of UVR. Sixteen healthy human subjects were given GTPs in combination with a vitamin complex. The preparation reduced UVR-induced erythema and inhibited UVR-mediated up-regulation of pro-inflammatory metabolites produced by 12-lipoxygenase (12-LOX). 12/15-LOX enzymatic balance plays a role in the pathogenesis of skin disorders as it regulates cell proliferation and apoptosis. The investigators concluded that the intake of GTPs resulted in the incorporation of catechin metabolites to human skin associated with a decrease of the 12-LOXE metabolite, possibly promoting protection against inflammation from sunburn and damage mediated by UVR (79). However, in a more recent human study (clinicaltrials.gov, NCT01032031) from the same group of investigators (122) using equal oral doses GTCs and vitamin C during the same period, no significant reduction in skin erythema, or leukocyte infiltration was found. Also, the investigators did not see alterations in the eicosanoid response to UVR.

Together with the controversial human results, there are significant limitations for the widespread use of GTPs preparations in preventing photodamage and photocarcinogenesis. GTPs are very sensitive to oxidation, rapidly losing their activity. Their half-life in the bloodstream is < 3 h (123). Another limitation is their poor solubility in lipid preparations, which significantly decreases its penetration through the skin, whereas it favors its absorption and oral uptake. To improve its penetration into the skin and its stability, GTPs can be mixed with non-toxic organic solvents, for example, oleic acid. However, it is necessary to further investigate the toxicity of GTPs at high doses (124).

### Cocoa extract

Cocoa (Chocolate) extracts are rich in polyphenols, mainly flavanols. Cocoa flavanols (CFs) have anti-oxidant properties, increasing the expression of HO-1 through of nuclear factor erythroid 2-related factor 2 (Nrf2) (125). Nrf2 is a regulator of cellular anti-oxidant responses that control the expression of genes encoding detoxifying proteins and anti-oxidant, such as HO-1. Cocoa procyanidins also inhibit MAPK activation and MMP expression (126). These mechanisms underlie their potential use in photoprotection and photocarcinogenesis (80, 125)*in vivo* studies showed that supplementation with cocoa powder in female albino hairless mice (Skh-1) attenuated UVB-induced skin wrinkling formation, regulating genes involved in extracellular dermal matrix degradation. Dietary cocoa decreased the expression of cathepsin G and improved the expression of Serpin B6c decreasing extracellular matrix (ECM) degradation (114).

In humans, oral consumption of CFs has potent anti-inflammatory, anti-oxidant, and photoprotective effects. In a clinical trial, two groups of healthy women, with Fitzpatrick's skin phototype II, undertook diets bearing high or low CFs for 12 weeks. A dietary beverage with cocoa rich in CF decreased the degree of erythema following irradiation with a solar light simulator (Δα valued decreased 68% from baseline). UV sensitivity did not change in the women with treatment with cocoa beverage bearing low doses of CFs (80).

In 2009, a double-blind study in 30 healthy subjects showed that consumption of a chocolate rich in flavonoids (HF) could prevent certain harmful effects of UV radiation in human skin, while conventional chocolate (LF) did not have this effect. MED after 12 weeks of HF chocolate treatment more than doubled, while it remained unaffected in subjects taking LF chocolate (81).

### *Polypodium leucotomos* extract (Fernblock®)

*Polypodium leucotomos* (PL) is a fern of the Polypodiaceae family, native to Central and South America. PL has been used in traditional medicine in those geographical areas for the treatment of skin conditions (1). A standardized aqueous extract of PL (PL/Fernblock®) made from leaves of the fern PL, rich in polyphenols, has been developed to exploit the photoprotective properties of ferns and to provide a steady phenolic content (87, 127). Our group has thoroughly investigated Fernblock® with regard to its anti-oxidant, anti-inflammatory, and immunomodulatory and tumor growth suppressive properties (1). Phenolic compounds identified in the aqueous extract Fernblock® are 4-hydroxybenzoic acid, 3,4-dihydroxybenzoic acid (protocatechuic acid), 4-hydroxy-3-methoxybenzoic acid (vanillic acid), 3,4-dihydroxycinnamic acid (caffeic acid), 4-hydroxycinnamic acid (p-coumaric),3-methoxy-4-hydroxycinnamic acid (ferulic acid), 4-hydroxycinnamoyl-quinic acid, and five chlorogenic acid isomers (128).

Ferulic and caffeic acids are the most potent anti-oxidants present in PL. Their apparent permeability shown in the Caco-2 cell *in vitro* model was 70–100%, similar to human post-oral administration absorption (127).

This extract was marketed in Europe in the year 2000, both in topical and oral forms, and is currently available in more than 26 countries, including the U.S. as a dietary supplement since 2006 (129). Its mechanisms of action and its success in clinical trials, and the increased social interest in natural substances such as polyphenols, have placed PL as an interesting photoprotective and anti-oxidant option (130, 131).

PL increases the ability of the endogenous anti-oxidant system. PL neutralizes superoxide anions, hydroxyl radicals, and lipoperoxides produced in the skin after exposure to UV and visible radiation (87, 88, 104, 127). The most significant differences between this extract and conventional anti-oxidants refer to its capacity as a superoxide anion scavenger. The majority of traditional anti-oxidants such as vitamin C, E, carotenoids are good quenchers of singlet oxygen; however, PL also exhibits excellent anti-oxidant properties against superoxide anion (87). In *in vitro* studies our group found that this extract was an efficient quencher of superoxide anion, with ~40 to 60% of the activity of SOD used as a positive control. Furthermore, it also inhibited lipid peroxidation (87, 88, 127). In addition to its anti-oxidant activity, PL shows promise in the prevention of photodamage and photocarcinogenesis because it enhances DNA repair and modulates the inflammatory and immune responses (1, 3, 129).

In the context of UVR-induced inflammation, our studies have revealed that orally administered PL prevented erythema in the UVR-treated human skin (82, 87). After oral administration of PL, the MED increased by 2.8 ± 0.59 fold (82). PL is also active on the skin as a photoprotector against PUVA-induced phototoxicity (83, 105). The basis of its anti-inflammatory properties could be its ability to abolish the expression of the TNF-α, iNOS (99), redox-sensitive transcriptional factors activator protein 1 (AP-1) and nuclear factor κB (NF-κB) (99). PL also decreases the expression of COX-2 and PGE2 (93). However, the effect of PL on AP-1 and NF-κB expression after exposure to solar simulated radiation (SSR) cannot be explained only by the anti-oxidant action of PL since treatment with a bona fide anti-oxidant does not decrease the expression of AP-1 and NF-κB in human keratinocytes subjected to SSR. *in vivo* experiments showed that COX-2 and PGE2 were overexpressed after exposure to UVR, but they both decreased in PL-fed mice (93). Other beneficial effects of oral PL included a decrease in UVR-induced infiltration of neutrophils and macrophages into the skin (93). Other studies showed a reduction in the levels of the inflammatory molecules, both in humans (83, 105) and in mice (101). These studies revealed an inhibition of mast cells and leukocyte extravasation in the irradiated area when PL is administered orally. These data complement *in vitro* studies using human PHA-stimulated peripheral blood mononuclear cells, which showed that PL decreased the production of IL-2, IFN-gamma, and TNF-alpha and completely inhibited the expression of the inflammatory cytokine IL-6. In the same experiments, the addition of PL increased IL-10 production (100). PL also inhibited apoptosis and cell death (89, 99) therefore preventing apoptosis/necrosis-triggered inflammation.

Moreover, orally administered PL inhibited UVR-mediated DNA damage and mutagenesis in humans and mice (83, 94, 99). PL exerted its effect by a double mechanism by preventing UV-induced accumulation of CPDs and reducing oxidative damage, with a reduction of 8-OH-dG. Also, even before UV irradiation oral PL decreased the levels of 8-OH-dG in a mouse model of Xeroderma pigmentosum (Xpc^+/−^), suggesting that oral PL relieves constitutive oxidative DNA damage (103). In this model, we found that PL inhibited expression of COX2 and accelerated CPD removal. In this regard, cells containing CPDs were detected immediately after UVB in both groups of animals, vehicle-, and PL-fed mice, confirming similar initial UVB damage. However, by 72 h, 54 ± 5% CPDs remained in vehicle-fed mice compared to only 31 ± 5% in PL-fed mice. These data indicate that PL increases the repair capacity rather than preventing the formation of thymine dimers. Also, we found that PL prevented UVR-mediated pro-oxidative DNA damage by quantifying cells containing 8-OH-dG, particularly in skin sections 6–24 h after exposure and also reduced the mutational burden by ~25% (93). Finally, oral PL decreased UVA-dependent mitochondrial DNA damage by reducing common deletions (CD) (95).

Regarding photo-immunosuppression PL is endowed with immunomodulatory properties acting as a photoimmunoprotective agent by different mechanisms. PL prevents UCA isomerization into from its *trans* to the *cis* isomer (103), which is a triggering event of skin immunosuppression. In turn, as the primary UV-absorbing chromophore in the skin, it prevents the expression of pro-inflammatory cytokines such as TNF-α (99). Also, PL prevents epidermal Langerhans cells (eLC) depletion produced by UV irradiation *in vivo* (82, 83, 101, 105). Multiple molecular mechanisms may underlie the improvement of survival of dendritic cells, including inhibition of UCA isomerization, as mentioned above (103), blockade of iNOS expression (99), and improvement of endogenous systemic anti-oxidant systems (89, 93, 101). Finally, orally administered PL also inhibited UVB radiation-induced immunosuppression in mice sensitized with oxazolone before UVR exposure and prevented inhibition of CHS (102).

Photoinmunosuppression is an essential area for preventing photocarcinogenesis. Our group has evaluated the possible protective action of oral PL in photocarcinogenesis. *in vitro* and *in vivo* studies showed that PL modulates the expression of molecules, transcription factors, and gene expression involved in photocarcinogenesis (1–3, 132). We found that PL delayed the onset of skin cancer in PL-treated hairless mice. PL also decreased the number of precancerous lesion in the surrounding non-tumoral skin of the same animal and elevated p53 expression levels (89). The delay in the initiation of photocarcinogenesis correlates with changes in the levels of several markers of oxidative stress in the skin and blood.

In this regard, PL-treated animals had increased anti-oxidant plasma activity, without changes in the levels of endogenous anti-oxidant enzymes (89). Oral PL also induced p53 overexpression in the Xeroderma pigmentosum Xpc^+/−^ mouse model that displays skin cancer highly comparable to mild human XP syndromes. PL-fed and UVB-irradiated, Xpc^+/−^ mice showed a 2–4 fold increase in the levels of total and pSer15 compared to vehicle-treated mice (93). DNA damage induced phosphorylation of p53 on Ser15 and Ser20. Phosphorylation inhibited the ability of negative regulator of p53, to bind p53, favoring both the activation and accumulation of p53 in response to DNA damage (133). In this experimental model, we found an inverse correlation between the increase of p53 and the decreased COX-2 levels, suggesting that oral PL treatment reduced UVR-induced COX-2 levels, at least in part, by activation of p53 (93). In agreement with the increased p53 expression, PL also decreased epidermal cell proliferation induced by UVR in human and experimental animals (83, 104). In clinical studies, we found that PL reduced the rate of proliferating epidermal cells induced by UVR (83). A recent study showed that PL decreases the number of cyclin D1- and PCNA-positive epidermal cells caused by UVR (134).

The ECM provides structural integrity to the tissue and is remodeled during skin aging/photoaging and cancer (115). *in vitro* experiments showed that PL directly inhibited the enzymatic activity and expression of MMPs in melanoma cells and fibroblasts. PL stimulated the expression of TIMPs in melanoma cells, reducing melanoma cell growth, and ECM remodeling (88, 115).

VL and infrared radiation (IR) also promote sun-induced skin damage (135, 136). The energy of IR and VL photons is much lower than that of UV photons. The most considerable part of solar IR radiation is IRA (IRA, wavelength 700–1,400 nm). IRA deeply penetrates into the human skin whereas IR B (IRB, wavelength 1,400–3,000 nm) and infrared radiation C (IRC, wavelength 3,000 nm−1 mm) only affect the upper layers (135). In human skin, IR irradiation generates heat and free radicals (136). IRA-induced photoaging, by generating mitochondrial ROS (137) followed by a cascade of intracellular events that leads to an increase of MMP-1 and MMP 9 without an increase of TIMP expression (138). Besides its effect on MMP, IRA also triggers infiltration of inflammatory cells into the skin (139). IRA, also, decreases the number of Langerhans cells, influences wound repair and alters the expression of transforming growth factor beta (TGB-β) (139). Regarding VL (400–700 nm), an early study from Pathak (140) indicated that VL produced an immediate darkening of the skin. VL contributed to ROS production in the skin (141) and induced DNA damage through the generation of ROS (142). VL exerts similar effects to UVR in the ECM. IR plus VL increased the expression of MMP-1 and MMP-9 and, in human skin *in vivo* lowered type I procollagen levels and recruited macrophages to the irradiated site (139).

We have also studied the possible effect of PL in preventing damage induced by IR plus VL. We found that PL was clinically effective in preventing the deleterious effects of infrared-visible IR–VL radiations (116). In a recent prospective clinical trial, volunteers received a combination of IR-VL (600 and 200 J/cm^2^, respectively). Gluteal biopsies were taken before and after irradiation. PL (960 mg/day) was administered orally for 21 days followed by another round of IR–VL radiation and biopsy. The results showed that MMP-1 was increased after VL-IR radiation concerning baseline in 71% of the patients, while the percentage of patients treated with PL was smaller (51%).

As we reported previously, PL reduces UVR-induced immunosuppression and mutagenesis. Patients with at least two AKs on the scalp underwent two sessions of PDT, separated by 1week. One group received PTD and oral PL treatment for 1 week after the last PDT session. Both treatment modalities PDT alone or PDT plus oral PL reduced the number of AK. However, supplementation with oral of PL increased the clearance rate and decreased the recurrence rate of AKs within 6 months, compared to PDT alone. Oral PL could be used as a supplementary agent in the treatment of field cancerization (110). We have also investigated the possible protective role of oral administration of PL in patients at risk of malignant melanoma (MM) and evaluated the influence of PL in the interaction between MC1R polymorphisms and the cyclin-dependent kinase (CDK) inhibitor 2A gene (*CDKN2A*) status with MED (84). 25–50% of familial MM relatives display a mutation in *CDKN2A* and variants in *MC1R* are common in the white population, conferring low to moderate risk to develop melanoma. In our trial, a total of 61 patients (25 with familial and/or multiple MM, 20 with sporadic MM, and 16 without a history of MM) were exposed to UVB radiation. Oral PL treatment increased by 30% the MED mean in all patients. Among patients with familial MM, those individuals with mutations in CDKN2A and/or MC1R had greater differences regarding the response to treatment with PL (84). According to these results, patients with higher UVR sensitivity (lower basal MED) would benefit the most with oral PL treatment. These results are intriguing and thus studies with longer-term PL administration in patients with a high risk of developing MM are needed to consolidate these data. Finally, PL also ameliorates the onset of the polymorphic light eruption, which is the most common photosensitivity condition of the skin (106–108).

Regarding the safety of oral treatment of PL, a recent study determined that capsules containing a carefully controlled extract of PL (Heliocare, IFC, Spain) (240 mg) have not produced severe adverse effects, after 2 months of treatment (143).

### Isoflavones

Isoflavones, one leading group of phytoestrogens, have the ability to act as topical photoprotectors. Oral photoprotection is not well-documented (144), and also not much information has been reported from studies in humans. Some isoflavones or isoflavone-rich compounds are genistein, equol, silymarin, quercetin, and apigenin.

#### Genistein

Genistein, an isoflavone obtained from fermented soy, coffee beans, and fava, is a potent tyrosine kinase inhibitor. Genistein has a robust anti-oxidant capability (145). Expression of the transcription factor Nrf2 is activated by oral treatment with genistein (146, 147). Oral genistein inhibited UVB-mediated skin photoaging and skin tumor formation in a rodent model (111).

#### Equol

Equol, a metabolite of the genistein analog daidzein, is enriched naturally with red clover (*Trifolium pratense*) (148). Although equol has yet to be used as an oral photo-protector, recent research indicates a high oral tolerance (149), suggesting that it may be appropriate for oral photoprotection. Topically, equol confered protection against photoaging (150) and also decreased tumorigenesis induced by UVR (149, 151).

#### Silymarin

Silymarin is a flavonoid derived from the milk thistle plant (Silybum marianum complex) that contains silybin, silydianin, and silychrisin. Its oral use in photoprotection has not been tested, whereas sylimarin topically applied confers photoprotection due to the amount of silybin in the preparation (152). Silymarin interferes with the bioavailability of other drugs (152) what may limit the use in oral photoprotection.

#### Quercetin

The polyphenol quercetin is the most abundant flavonoid, and it is found in fruits, vegetables, tea, and wine. Quercetin is a potent anti-oxidant, and it works as a topical photoprotector (153), but until now it has not been evaluated in oral photoprotection. Similar to silymarin, it can alter the bioavailability of other drugs (154).

#### Apigenin

Apigenin is a flavonoid found in several fruits, vegetables including onions, parsley, and sweet red peppers as well as tea. Several studies conducted over the past years have reported its potential as an anti-oxidant, anti-inflammatory, and anti-cancer compound (155). Topically, apigenin decreased tumor emergence after exposure to UVR in a rodent model. This effect may have been caused, at least in part, by inhibition of both COX2 and the mammalian target of rapamycin signaling pathway (156–158). However, its usefulness as an oral photoprotector has yet to be addressed.

### Pomegranate (*Punica granatum*, fam. punicaceae)

The anti-oxidant activity of pomegranate juice is very high, e.g., higher than that of red wine and green tea due to its polyphenolic content, which includes anthocyanidins and catechins and tannins (159). As an oral photoprotector, the Mukhtar group has described the efficacy of pomegranate polyphenols in the prevention of photocarcinogenesis in mice irradiated with UVB (90, 160, 161). These authors claimed that pomegranate fruit extract inhibited the expression of COX-2 and iNOS, as well as the expression of cyclin D1 in mouse skin after UVB irradiation. Also, this extract decreased the expression of MMP2, 3, and 9 in the skin of the mouse model (90, 160, 161).

### Citrus plus rosemary extract

Citrus contains a large amount of flavonoids, and rosemary is rich in polyphenols and diterpenes. In humans, oral administration of a combination of citrus and rosemary extracts decreased sensitivity to erythema induced by UVR, as quantified by an increased MED that after 8 weeks of treatment ranged from 34% in Perez-Sanchez's study (85) to 29.8% in Nobile's study (86).

### Resveratrol

Resveratrol is a polyphenolic phytoalexin stilbenoid found in the peels and seeds of grapes as well as red wine. The effect of resveratrol as a topical photoprotector is well documented (162). Regarding its action as an oral photoprotector in a p53-sensitive mouse tumor model, the administration of oral resveratrol decreased the tumorigenesis mediated by UVR (112) through the modulation of TGF-beta (112) and NF-kB (163). Also, resveratrol may have the potential to stimulates the response to radiation therapies (164).

### Forskolin

The diterpenoid forskolin (FSK) is obtained from the root cork of the Indian coleus (*Coleus forskohlii*). It is a classical activator of the adenylate cyclase enzyme resulting in elevated levels of cyclic adenosine monophosphate (cAMP). A recent study addressing the effect of FSK in UVR-mediated photodamage reported that FSK accelerated the removal rate of UVR-induced photolesions *in vitro* and *in vivo* (96). Topical application of forskolin also restored pigmentation UVR-independent in an MC1R-defective fair-skinned animal model (165).

Cutaneous melanocortin one receptor (MC1R) initiates multiple protective actions against deleterious effects of UVR, including melanin production. These actions are mediated by the activation of adenylyl cyclase and cAMP. Eumelanization by FSK is thought to occur by direct activation of adenylyl cyclase in melanocytes and up-regulation of melanocyte cAMP levels. Polymorphisms of MC1R induce a fair-skinned, sun-sensitive, and cancer-prone phenotype. Mice bearing inactivating mutations in this gene (Mc1re/e) lacked the ability to generate cAMP in response to MSH. In those mice, cutaneous application of FSK promoted DNA repair in response to UVR photodamage. The defect of these transgenic mice underlies in an inability to remove CPD induced by the UVR, which is significantly increased by FSK to levels comparable to those of Mc1r wild-type mice (166). FSK also exerted its photoprotective effect by increasing epithelial thickening due to increased keratinocyte proliferation in a cAMP-dependent manner (167). *in vitro*, FSK has also demonstrated a photoprotective impact by increasing epithelial thickness, favoring the proliferation of keratinocytes in a cAMP-dependent way (113). *in vitro*, FSK inhibited keratinocyte apoptosis induced by UVR, reducing sunburn cells count. Interestingly, melanin content levels were independent of FSK treatment, showing that the protection against apoptosis was not the result of an increase in melanin levels (168).

FSK also promotes cellular growth to repair skin photodamage. Specifically, FSK improves NER after exposure to UVR; however, this effect only appeared in growing skin cells. When cells were cultured at low density, FSK stimulated cAMP responsive element binding (CREB) phosphorylation, which is a marker of PKA activation, producing a significant increase in the activity of NER compared to the control. These findings indicate that cell growth is critical for FSK to improve NER function and suggest that cell growth conditions should be considered as a variable while evaluating the FSK efficacy in inhibiting UVR-induced photodamage (96). FSK has been used orally for non-skin-related therapeutic uses, but not in skin disease (168).

## Evaluating oral photoprotection

The classical model of evaluation of topical photoprotectors includes SPF assessment, based on prevention of erythema. Another useful indicator is the erythema protection scale, which measures skin reddening due to inflammation. However, oral photoprotectors are not very effective in reducing erythema and thus cannot be evaluated using SPF and erythema protection factor scales. These reagents need to be measured according to other parameters, which include:

### Anti-oxidant activity

Approaches include irradiation of keratinocytes with UVB followed by detection of T-T dimers and sunburn cells and have the potential to become a gold standard to gauge the photoprotective ability of new oral compounds. An additional test could include measuring anti-oxidant potential *in vitro*. The main drawback is that this approach does not allow to extrapolate the effect of oral administration directly. In general, the previous methods always need to be complemented with studies on oral toxicity, metabolic disposition, and careful assessment of the pharmacodynamics and pharmacokinetics of an oral agent.

### Anti-mutagenic activity

This approach is currently applied in nonhuman models, and it is based on the ability of the compound(s) under analysis to prevent mutations in critical genes involved in photocarcinogenesis, e.g., p53 (169). Two common reference assays employ mouse bone marrow-derived erythrocytes and the TA100 strain of *Salmonella typhimurium*, which is histidine-dependent.

### Photoimmunoprotection

A useful parameter includes measuring the effect of the oral intake of the compound of interest on UVR-induced inhibition of contact or delayed-type hypersensitivity responses. This measure can be done in one or two ways: (i) a single sub-erythemal dose of UV radiation. This protocol enables a more direct comparison with the SPF parameter used to evaluate topical sunscreens. However, this approach requires a large cohort of healthy volunteers. This renders this approach not particularly cost-effective (170); (ii) using a pre-sensitization screening with chemical irritants. A significant problem with this approach is that the chemical sensitization is not directly comparable to damage induced by UVR. However, it brings a reasonable estimate of the immunomodulatory properties of the treatment.

The practical aspects of the use and prescription of oral photoprotectors need to be evaluated by available information on biodisposition, efficiency, and safety. A gold standard is still lacking in this regard, but one positive is the overall low toxicity of these agents (after all, many of them are part of nutrients). However, specific aspects, e.g., known allergies, must be taken into account when using or prescribing these approaches.

## Future perspectives

Oral supplementation aims at countering the long-term effects of sun exposure. Many of these effects are related to immunosuppression, chronic inflammation, and photocarcinogenesis. The current view of many research groups, including ours, is that this developing field needs the establishment of strong standards to enable a rigorous assessment of the effectiveness of oral photoprotection. These need to include measurements on anti-oxidant activity, anti-mutagenic capability, and anti-immunosuppressive function. The FDA, EMA, and other regulatory agencies around the world need to become involved in the establishment of gold standards and regulate the research on the growing landscape of new substances and combinations of substances that will likely change the field of photoprotection in years to come.

## Author contributions

SG conceived the review and wrote the sections of *Evaluating Oral Photoprotection* and *Futures Perspectives*. CP, NP, YG, and AJ have contributed to the different sections of the manuscript and have read and corrected the entire manuscript. SG has overseen the integration of the entire manuscript and has read and corrected the entire manuscript.

### Conflict of interest statement

The authors declare that the research was conducted in the absence of any commercial or financial relationships that could be construed as a potential conflict of interest. The reviewer FL and the handling Editor declared their shared affiliation.
